# Detection of Chilling Injury in Pickling Cucumbers Using Dual-Band Chlorophyll Fluorescence Imaging

**DOI:** 10.3390/foods10051094

**Published:** 2021-05-14

**Authors:** Yuzhen Lu, Renfu Lu

**Affiliations:** 1 Department of Agricultural and Biological Engineering, Mississippi State University, Starkville, MS 39762, USA; 2 United States Department of Agriculture Agricultural Research Service, East Lansing, MI 48824, USA; renfu.lu@usda.gov

**Keywords:** pickling cucumber, chilling injury, chlorophyll fluorescence imaging, machine learning

## Abstract

Pickling cucumbers are susceptible to chilling injury (CI) during postharvest refrigerated storage, which would result in quality degradation and economic loss. It is, thus, desirable to remove the defective fruit before they are marketed as fresh products or processed into pickled products. Chlorophyll fluorescence is sensitive to CI in green fruits, because exposure to chilling temperatures can induce detectable alterations in chlorophylls of tissues. This study evaluated the feasibility of using a dual-band chlorophyll fluorescence imaging (CFI) technique for detecting CI-affected pickling cucumbers. Chlorophyll fluorescence images at 675 nm and 750 nm were acquired from pickling cucumbers under the excitation of ultraviolet-blue light. The raw images were processed for vignetting corrections through bi-dimensional empirical mode decomposition and subsequent image reconstruction. The fluorescence images were effective for ascertaining CI-affected tissues, which appeared as dark areas in the images. Support vector machine models were developed for classifying pickling cucumbers into two or three classes using the features extracted from the fluorescence images. Fusing the features of fluorescence images at 675 nm and 750 nm resulted in overall accuracies of 96.9% and 91.2% for two-class (normal and injured) and three-class (normal, mildly and severely injured) classification, respectively, which are statistically significantly better than those obtained using the features at a single wavelength, especially for the three-class classification. Furthermore, a subset of features, selected based on the neighborhood component feature selection technique, achieved the highest accuracies of 97.4% and 91.3% for the two-class and three-class classification, respectively. This study demonstrated that dual-band CFI is an effective modality for CI detection in pickling cucumbers.

## 1. Introduction

In the U.S., cucumber production falls into two types, i.e., fresh-market and processing for pickled products. Pickling cucumbers account for more than half of the total production of cucumbers in the nation, valued at $144 million at the farmgate in 2019 [1]. Pickling cucumbers are characterized by being much shorter than fresh types, with lighter colored skin and more prominent warts [2]. In addition to making pickles, pickling cucumbers can also be sold fresh for immediate consumption. Harvested cucumbers, either processing or fresh-market, are routinely stored under reduced or refrigerated temperatures (e.g., 10 °C or below) to maintain fruit quality attributes (e.g., firm texture and green color). However, cucumbers are chilling-sensitive, which may develop symptoms of chilling injury (CI) after prolonged exposure to low temperatures at 7–13 °C [3]. CI results from various physiological and biochemical dysfunctions including cell membrane degradation, metabolite leakage and pigments degradation. Typical CI symptoms are surface pitting, water-soaked areas, discoloration and accelerated decay [4,5], depending on severity and fruit variety [6,7], and these symptoms may appear or develop rapidly when the chilled fruit are returned to a higher temperature. The presence of CI is an important quality defect for cucumbers, causing downgrading and economic loss. The U.S. Standards [8,9] for pickling cucumbers grade the fruit with scorable freeze damage into culls, implying an outright product rejection. Hence, there is a need for inspection and sorting of pickling cucumbers for CI, so as to remove the injured fruit during postharvest handling.

Currently, no automated or rapid methods are being commercially used for CI detection of pickling cucumbers. Development of non-destructive optical techniques for CI detection would be beneficial for enhancing quality inspection of pickling cucumbers and pickled products. Hyperspectral imaging has been extensively researched for quality and safety assessment of horticultural products [10,11]. Cheng et al. (2004) applied hyperspectral reflectance imaging for CI detection of cucumbers, and achieved a detection accuracy of 93.3% for injured fruit by combing principal component analysis (PCA) and Fisher’s linear discriminant [12]. In similar studies [13,14], large spectral differences between sound- and chilling-injured skins of cucumbers were observed in the wavelength region of 700–850 nm, and the band reflectance ratio of R811 nm/R756 nm could detect the injured skin with an accuracy of over 90%. However, the authors also pointed out the difficulty in detecting CI at the early stage of 0 to 2 days of post-chilling storage, due to insignificant manifestation of symptoms. More recently, Cen et al. (2016) applied a hyperspectral imaging system in integrated reflectance (500–675 nm) and transmittance (675–1000 nm) mode for CI detection of cucumbers [15]. The overall accuracies of 100% and 91.6% were obtained for two-class and three-class classification for cucumber fruit, respectively, by extracting features from band-ratio images followed by support vector machine modeling. However, hyperspectral imaging still faces critical challenges in real-time image acquisition and processing of high-volume image data as well as high hardware costs for practical online inspection of food products [10].

Fluorescence techniques provide a sensitive sensing modality for detecting stress-induced defects or disorders for horticultural products [16,17], which is complementary to reflectance and transmittance techniques. Green-skinned fruit, when excited by ultraviolet (UV) and/or blue light, emit longer-wavelength fluorescence due to the absorption of chlorophyll components. Photosynthesis can be markedly reduced in CI-sensitive plants when exposed to chilling temperatures [5,18], which suggests that chlorophyll fluorescence is potentially useful for CI detection. Abbott and Massie (1985) first applied refreshed delayed light emission (DLE), also termed as delayed chlorophyll fluorescence, for the discrimination of CI-affected from healthy cucumbers [19]. However, DLE measurements required dark equilibrium of at least one hour, which would not be suitable for practical application. The chlorophyll fluorescence parameter Fv/Fm (i.e., the ratio of variable chlorophyll fluorescence to maximal chlorophyll fluorescence intensity) is an important measure of the maximum quantum efficiency of photosystem II photochemistry, providing an indicator of photosynthetic performance and physiological conditions of plants. A study by van Kooten et al. (1992) reported a dramatic decrease in Fv/Fm, implying suppressed photochemical activity, for cucumbers stored at chilling temperatures of 4 °C and 7 °C, compared to those at 10 °C and 13 °C [20]. Similar findings were also reported for cucumbers stored at 1 °C versus 4 °C [7], and for fruit hydro-cooled with water at 1.5 °C or 3.5 °C versus 6–10.5 °C [21]. These studies demonstrated the feasibility of using chlorophyll fluorescence for detecting CI in cucumbers, but they were only performed as point measurements using a fluorometer, which are inadequate for characterizing the spatial features of the defect.

Compared to conventional point-measurement fluorescence techniques, fluorescence imaging enables acquiring two-dimensional fluorescence signals of samples. It allows to visualize the spatial fluorescence patterns on samples and is, thus, more suitable for detecting the localized defects [16,22]. Nedbal et al. (2000) used a kinetic chlorophyll fluorescence imaging (CFI) system for the detection of mold infections [23]. The diseased area exhibited very high F_0_ (minimal chlorophyll fluorescence intensity) and low F_v_ (variable fluorescence) and F_v_/F_m_, and the fluorescence image of F_0_/F_v_ (the map of the ratio of the two fluorescence parameters F_0_ and F_v_) allowed discriminating between diseased and healthy areas. Obenland and Neipp (2005) reported that the chlorophyll fluorescence images of F_0_ and F_m_ were effective for detecting hot water-induced skin damage for lemons that had been immersed in 55 °C water for 5 min [24]. Fluorescence imaging for other fluorophores has also been exploited for defect detection of horticultural commodities. Slaughter et al. (2008) detected the freeze damage of oranges by acquiring color images of the yellow-fluorescent emissions of peel oil constituents [25]. Kondo et al. (2009) conducted a similar study on the detection of rotten citrus fruit and examined the fluorescent substances [26]. Researchers also developed fluorescence imaging systems in multispectral [27,28] or hyperspectral [29] mode for detecting foreign contaminants on horticultural products. Until recently, however, no studies have been reported on using fluorescence imaging for detecting CI in pickling cucumbers. Lu and Lu (2020) identified two wavebands at 675 nm and 750 nm for imaging chlorophyll fluorescence of cucumbers under UV-blue light excitation and developed an algorithmic method for correcting the vignetting of fluorescence images [30].

This study extended from our previous work [30] to develop discriminative models based on chlorophyll fluorescence images for detecting CI-affected pickling cucumbers. The specific objectives of the study were to: (1) acquire dual-band chlorophyll fluorescence images at 675 nm and 750 nm from pickling cucumbers after chilling treatments; (2) examine the CI symptoms of pickling cucumbers based on visual inspection of chilled fruit and the acquired fluorescence images; (3) develop support vector machine (SVM) models based on the features extracted from the fluorescence images for classifying normal and CI-affected fruit.

## 2. Materials and Methods

### 2.1. Pickling Cucumbers

Pickling cucumbers of the ‘Vlaspick’ variety were hand-picked from a commercial farm in Ravenna, Michigan, during the 2018 harvest season. The freshly harvested cucumbers were first submerged in the wash tank and rubbed clean using soft sponge. Only cucumbers that were free of visual defects and of commercial size three [38–51 mm (1.5–2 inch) in diameter] were selected for this study.

**Figure 1:**
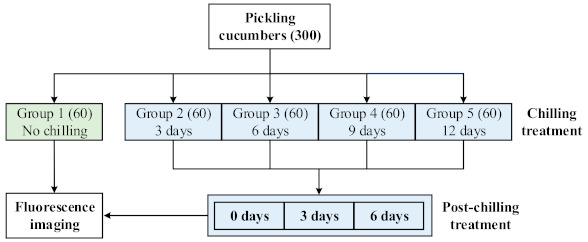
The groups of pickling cucumbers subjected to different treatments. The chilling and post-chilling (i.e., removing samples from cold storage to room temperature) treatments for different days are to induce varying degrees of chilling injury damage.

As shown in Figure 1, a total of 300 pickling cucumbers were randomly divided into 5 groups of 60 samples each; one group served as control and all other groups were subjected to chilling treatments at 5 °C (with ~80% relative humidity) in a temperature-controlled and darkened storage room for 3, 6, 9 and 12 days, respectively, to induce varying degrees of CI damage. Fluorescence images were first acquired from the non-chilled control group. Afterwards, each group of chilled samples were imaged on the 0th, 3rd and 6th day (post-chilling treatments), respectively, after they had been removed from cold storage to room temperature (~22 °C). The post-chilling treatment was to allow visual symptoms of CI to develop and manifest. It was noted that as post-chilling treatments proceeded, some chilled cucumbers were seriously decayed, and others had been affected with visible yellow spots on the fruit skin (for unknown reasons). These samples were eliminated from the sample set for further imaging. Finally, fluorescence images were acquired from a total set of 618 samples (the same samples would be counted multiple times as they were imaged on multiple dates). During the chilling and post-chilling treatments, each chilling group of cucumbers were stored in a plastic bag punched with small holes to allow for air circulation, while maintaining high humidity.

**Figure 2:**
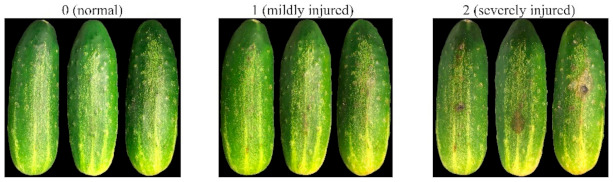
Examples of pickling cucumbers with three chilling injury levels.

Based on the visual inspection of CI symptoms for the chilled fruit after image acquisition for each date, the pickling cucumbers were categorized into three injury levels: 0 (normal), 1 (mildly injured) and 2 (severely injury). Figure 2 shows the photographs of typical pickling cucumbers with three CI levels. Normal cucumbers included the control samples and those subjected to chilling but without noticeable CI symptoms. A cucumber would be categorized as mildly injured when the fruit developed slight surface lesions with each spot being less than 5 mm and exhibited slight discoloration but no visible skin pitting and no decay or rot. Severely injured cucumbers are those having enlarged surface lesions, water-soaked spots with noticeable surface pitting or tissue decay. As a result, the final set of 618 samples comprised 180 normal, 268 mildly injured and 170 severely injured samples.

### 2.2. Chlorophyll Fluorescence Imaging

**Figure 3:**
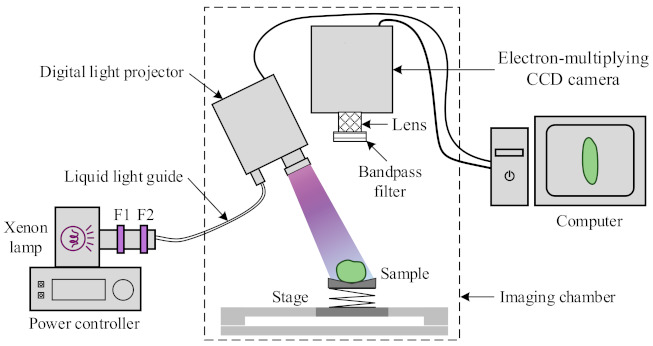
Schematic of a chlorophyll fluorescence imaging system for detecting chilling injury on pickling cucumbers, where F1 and F2, representing a bandpass (400 ± 70 nm) and a lowpass (<470 nm) filter, respectively, were used in combination with a Xenon lamp for generating ultraviolet-blue excitation light for chlorophyll fluorescence.

Figure 3 shows the schematic of an in-house assembled chlorophyll fluorescence imaging system for CI detection of pickling cucumbers. A 300-W broadband Xenon short-arc lamp (Model UXL-302-O, USHIO, Cypress, CA, USA), in conjunction with a radiometric power supply controller (Spectra-Physics, Stratford, CT, USA), was used as a light source for fluorescence excitation. Two filters, one a bandpass filter centered at 400 nm with a bandwidth of 70 nm (Corion, Holliston, MA, USA) and the other a lowpass filter with the cut-off wavelength of 470 nm (Edmund Optics, Barrington, NJ, USA), were placed in front of the light source housing to generate UV-blue illumination. The light output was channeled into a digital light projector (DL*i* CEL5500-Fiber, Digital Light Innovations, Austin, TX, USA) via a UV-visible light efficient liquid light guide (Model #77628, Newport Corp., Irvine, CA, USA) to provide uniform illumination over the sample. A back-illuminated electron-multiplying CCD camera with a resolution of 1024 × 1024 pixels (PhotonMax:1024B, air-cooled, Princeton Instruments, Trenton, NJ, USA), attached with a 35-mm C-mount fixed focal length lens (Stock #67-716, Edmund Optics Inc., Barrington, NJ, USA), was used for acquiring fluorescence images. Based on an earlier study [30], two bandpass filters centered at 675 nm and 750 nm (Stock #86-954 and #84-788, Edmund Optics Inc., Barrington, NJ, USA) with a bandwidth of 50 nm were selected and rotated in turn, attached in front of the focusing lens, for capturing the chlorophylls fluorescence emissions of cucumbers in the red and far-red regions, respectively. The CFI system was operated in an enclosed chamber to prevent interference from ambient light.

During the imaging, the cucumber sample was placed in random axial orientation on a V-groove holder on top of a vertically adjustable stage (Model #271, Newport Corp., Irvine, CA, USA) (Figure 3), with the chilling-injured areas (if present) of the fruit facing upwards to the camera. Only one orientation of each sample was imaged in this study. After the image acquisition had been completed, a color image of the sample in the same orientation was captured using a digital color camera, which was later used for visual assessment of chilling damage of the fruit (Section 2.1). Image acquisitions were performed using a custom-developed graphical user interface in LabVIEW 2016 (National Instruments, Austin, TX, USA) for camera control, with on-chip 2 × 2 binning and an exposure time of 1 s.

### 2.3. Image Processing

**Figure 4:**
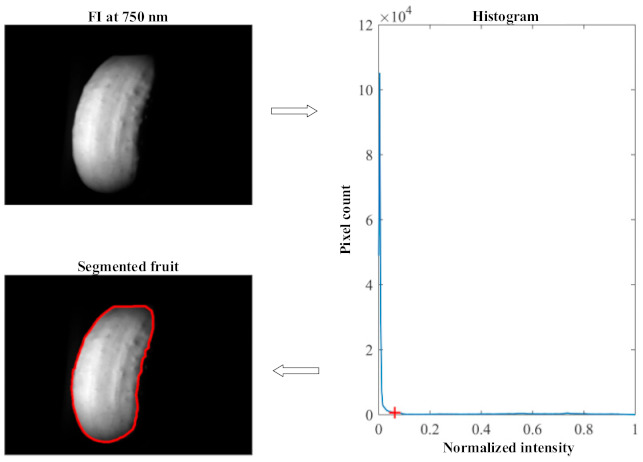
Fruit segmentation from the background by automatically thresholding the histogram of a fluorescence image (FI) at 750 nm. The red-cross marker in the histogram indicates the threshold determined by a unimodal thresholding technique [31], and the area enclosed by the red line is the resultant fruit segment.

The image processing consisted of two major steps, i.e., fruit segmentation and image vignetting correction. Segmentation of the fruit from its background could be readily achieved by applying a global threshold to the acquired fluorescence images, given the fact that the background had negligible fluorescence emissions at the wavelengths of interest (i.e., 675 nm and 750 nm). Fluorescence images at 750 nm had better visual contrast between the fruit and background than those at 675 nm, and hence, they were used for fruit segmentation. It was found that the histogram of fluorescence images was predominantly unimodal (Figure 4), with the single main peak due to the background located at the lower-intensity end. Hence, a unimodal-histogram-based automatic thresholding technique [31] was employed to segment the fruit from the background. Figure 3 illustrates the fruit segmentation process.

**Figure 5:**
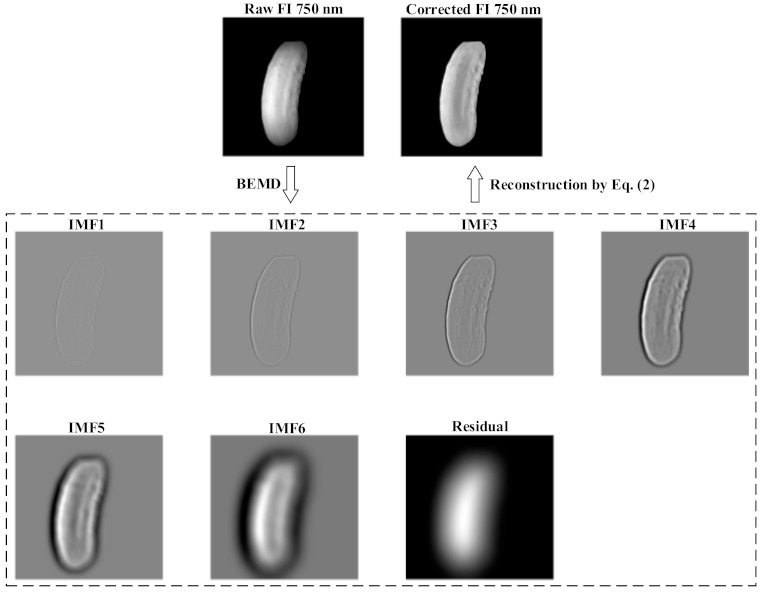
An example of bi-dimensional empirical mode decomposition (BEMD) of a raw fluorescence image (FI) at 750 nm into six intrinsic mode function (IMF) images, and vignetting correction of the FI image through image reconstruction defined by Equation (2).

The raw fluorescence images suffered from vignetting artifacts characterized by signal intensity fading out towards the image periphery. Such artifacts could be due to the combined effect of uneven illumination, the non-flat geometry of samples and the imperfection of imaging optics. The conventional method for correcting the vignetting of fluorescence images is to perform field corrections by acquiring fluorescence images from a flat fluorescent reference target that has sufficient fluorescence emissions at the wavebands of interest [32]. Preparing such a fluorescent reference is, however, costly and may not always be feasible. In this study, a new algorithmic method based on bi-dimensional empirical mode decomposition (BEMD) was applied for vignetting correction of fluorescence images [33]. For a raw fluorescence image (*FI*), applying BEMD to the image leads to a representation as follows:

(1) $$FI=\sum_{i=1}^{n}IMF_{i}+R$$

where *IMF_i_* is the *i*th intrinsic mode function (IMF) image, which carries the information at decreasing spatial scales for the original image, as *i* increases from 1 to *n*, and *R* is a residual image representing the basic trend (information at the coarsest spatial scale) of the original image. Since vignetting is a slowly varying background component, it can be assumed that *R* contains the information due to vignetting. Subsequently, a vignetting-corrected fluorescence image (*FI*_corr_) can be calculated through image reconstruction as follows:

(2) $$FI_{\mathrm{corr}}=\left(FI+\sum_{i=2}^{k}IMF_{i}-IMF_{1}\right)/R,\;k<n$$

where *n* and *k* are two tunable parameters that may affect the quality of BEMD and image correction; in this study, the two parameters were empirically set to 6 and 3, respectively. To eliminate high-frequency noise, the first IMF was excluded in the reconstruction. Figure 4 illustrates the process of BEMD and image vignetting correction. Compared to the original raw image, the BEMD-corrected image became more uniform in the intensity distribution across the entire surface of the fruit (Figure 5). A detailed description of the principle and implementation of BEMD is given in [33] and references therein.

### 2.4. Machine Learning

**Figure 6:**
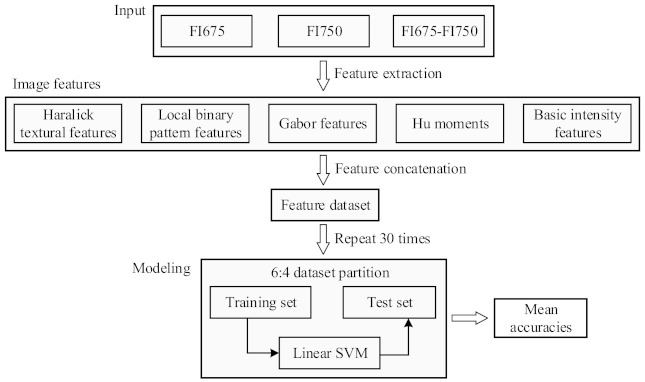
Machine learning procedures for building classification models using the features extracted from the fluorescence images. FI675 and FI750 denote chlorophyll fluorescent images at 675 nm and 750 nm, respectively, and SVM is short for support vector machine.

Figure 6 shows the flowchart of the machine learning procedures to build classification models for classifying pickling cucumbers. The models were developed for three types of image input respectively, i.e., the fluorescence images for single wavelengths of 675 nm (FI675) and 750 nm (FI750) and their combination (FI675-FI750). This would allow examining the efficacy of using single-wavelength images for CI detection. In each of the three scenarios, different types of textural and intensity features were extracted for each sample, and then concatenated together into a single feature vector. In this study, the extracted features included 28 Haralick features, 59 local binary pattern (LBP) features, 67 Gabor features, 6 basic intensity features and 7 Hu-moments features, thus forming a complete set of 167 features per sample for the image input of either FI675 or FI750, and 334 (i.e., 2 × 167) features for the input of FI675-FI750. The input FI675-FI750, which is a fusion or concatenation of the features from FI675 and FI750, is expected to obtain better accuracies by taking the advantage of an enriched set of discriminatory information [34]. A detailed description of the extracted features and their computations and discriminative power on the classification of normal and defective apples can be found in our earlier study [35,36] and references therein. Prior to developing classification models, the feature dataset was feature-wise normalized to have zero mean and a unit variance, and it was then randomly partitioned into training and test sets according to a ratio of 6:4.

Support vector machine (SVM) is commonly used for various classification tasks due to its high accuracy and the ability to model high-dimensional, complex data. Compared to other advanced machine learning algorithms like convolutional neural networks, SVM requires less computational resources and can be sufficiently optimized in a timely manner. There are different versions of SVM depending on whether and how kernel tricks (e.g., linear and non-linear kernels) are implemented for solving linear or nonlinear problems [37]. In this study, linear soft-margin SVM [38] was chosen for the modeling tasks because of the simplicity of hyperparameter optimization while being able to attain decent accuracies. Two types of models were built in this study for two-class (“normal” and “injured”) and three-class (“normal”, “mildly injured” and “severely injured”) classification of cucumber fruit, respectively. In model training, a regularization hyper-parameter controlling the tradeoff between maximizing the margin and minimizing misclassifications [39] was optimized through five-fold cross validation, in which the hyper-parameter was sampled over a range of [1 × 10^−4^, 1 × 10^4^]. Because of the randomness of feature dataset partitions (Figure 6), it would be desirable to repeat the modeling procedures multiple times for obtaining a reliable estimate of classification performance. In this study, 30 training replicates were conducted, and the mean test accuracies were computed for model comparison.

To reduce the model complexity while boosting its accuracy, feature selection or reduction was done for each of the two-class and three-class modeling scenarios, where the best test accuracy was obtained with a full set of features. No extensive feature selection efforts for all the modeling scenarios were made, as it would be beyond the scope of the present research. Instead, a feature selection method based on neighborhood component analysis (NCA) [40] was used to rank the features in a descending order of importance. The NCA method selects the feature subset by optimizing nearest neighbor classification in which a regularization term is introduced in the objective function of classification accuracy to alleviate potential overfitting [40]. A set of SVM models were built by incrementally including one more feature from a subset of the top 100 features ranked by importance, which were found sufficient based on preliminary experiments. To ensure fair comparisons with full-feature-based SVM models, the feature selection-based modeling was performed with 30 replicates as well.

### 2.5. Performance Metrics

The classification performance of SVM models was evaluated in terms of true positive (TP), true negative (TN), and overall accuracy (ACC) [41]. The TP and TN rates were calculated as the percentages of correctly classified defective and normal samples, respectively, with respect to the total samples of the associated class. The ACC was the percentage of all correctly classified samples, regardless of classes, with respect to all the tested samples. In the case of the three-class classification, TN rates were calculated for both the “mildly injured” and “severely injured” classes, and denoted by TN1 and TN2, respectively. Furthermore, statistical comparisons were conducted on the ACC values among the three image inputs (i.e., FI675, FI750 and FI650-FI750), and between the models using a full set of features and subset of selected features, using Fisher’s least significance difference procedure combined with the ANOVA test at the 5% significance level.

### 2.6. Software

All the analyses for image preprocessing, feature extraction and machine learning were performed in Matlab R2018b (The Mathworks, Inc., Natick, MA, USA).

## 3. Results and Discussion

### 3.1. Chilling Injury Symptoms

**Figure 7:**
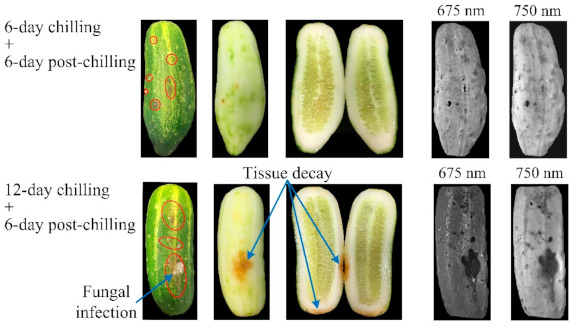
Color photos and chlorophyll fluorescence images for two chilling-injured pickling cucumbers (one sample in each row). The areas enclosed by the red ellipses indicate the noticeable tissue lesions.

Figure 7 shows the color photos and chlorophyll fluorescence images of two chilled pickling cucumbers with noticeable CI symptoms. CI was visually manifested as skin pitting, water-soaked spots, browning and tissue decay, and in the advanced cases of decay, fungi had grown on the fruit skin. Examination of the injured samples, after having been peeled and cut open, revealed that the injured tissues were mainly confined to the areas at or close to the fruit surface. This is because CI is developed as a result of destruction and collapse of hypodermal and epidermal cells that are situated tens or hundreds of micrometers below the fruit surface [3,42]. The fluorescence images were effective for visualizing the injured tissues that appeared as darkened spots or patches in the images, even when only slight external symptoms had developed. Compared to the fluorescence images at 750 nm, the images at 675 nm had sharper appearance, as was observed in an earlier study [30], but had greatly reduced signal intensities, since fluorescence emissions around 675 nm could be strongly re-absorbed by chlorophylls [43].

### 3.2. Classification

**Figure 8:**
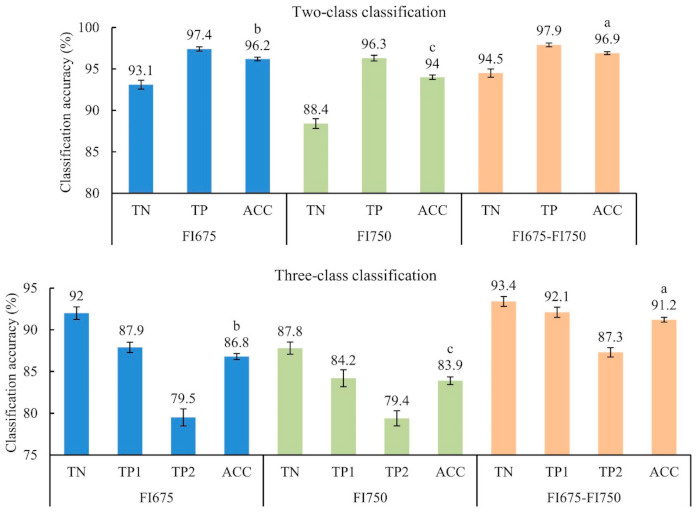
Two-class and three-class classification for chilling injury detection of pickling cucumbers. TN, TP and ACC represent true negative, true positive and overall accuracy, respectively, and TP1 and TP2 represent the true positives for mildly and severely chilled cucumbers, respectively. FI675, FI750 and FI675-FI750 represent the three types of the input of fluorescence images at 675 nm, 750 nm and their combination, respectively. Error bars in the chart indicate the corresponding standard errors for the accuracies over 30 modeling replicates. Different letters in the ACC accuracy indicate statistical difference at the 5% significance level.

Figure 8 (top panel) shows the average classification results from two-class (normal and injured) SVM models (for 30 modeling replicates) for the test datasets. The injured class was the combination of the samples that were categorized as “mildly injured” and “severely injured” (Section 2.1). Among the three types of image input, FI675-FI750 achieved the best overall classification accuracy of 96.9%, which was statistically significantly higher than those obtained by FI675 and FI750. This result should have been expected, since FI675-FI750 integrated or fused the features from FI675 and FI750, which could be complementary in ascertaining CI with varied symptoms and lead to enhanced discriminating power for CI detection. The single-band FI675 and FI750 also achieved comparably high accuracies, and the former performed significantly better than the latter, indicating that the wavelength 675 nm was more informative than 750 nm in detecting CI. It is noted that in all the three modeling scenarios, the TP was consistently higher than the TN, which was likely due to the unbalanced dataset (180 normal vs. 438 injured samples) in this study. These results demonstrated that the dual-band CFI technique is a useful tool for discriminating CI-affected from normal pickling cucumbers.

Figure 8 (bottom panel) depicts the three-class classification results. The three-class SVM models (for 30 modeling replicates as well) resulted in substantial reductions of ACC by 9.8%, 10.1% and 5.7% for the input of FI675, FI750 and FI675-FI750, respectively, compared to the two-class classification. The deterioration in ACC was due to increased errors in classifying the “mildly injured” and “severely injured” samples (i.e., decreased TP1 and TP2 rates). The majority of misclassifications were caused by incorrectly classifying one of the two CI classes into the other, especially for the “severely injured” class. The subjective scoring in defining the two ground-truth CI classes could also have contributed to the diminished accuracy. Despite reduced accuracies, FI675-FI750 still performed the best in terms of ACC, followed by FI675 and FI750, and their accuracy differences were statistically significant as in two-class modeling. Moreover, the improvements in ACC due to the ensemble FI675-FI750, that is, 4.4% and 7.3% relative to FI675 and FI750, respectively, were noticeably larger in the three-class classification than those (0.7% and 2.9%) in the two-class classification. These results are expected since in the two-class classification, the individual feature set, especially at 675 nm, already had high discriminative power.

**Figure 9:**
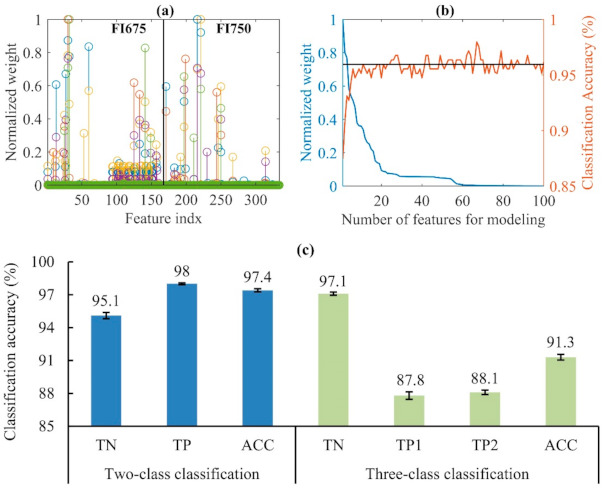
(**a**) Normalized feature weights obtained by neighborhood component analysis feature selection for one modeling replicate of two-class classification based on FI675-FI750, (**b**) the top 100 ranked features from (**a**) and the corresponding classification accuracies, where the black horizontal line indicates the classification accuracy using a full set of features, and (**c**) two-class and three-class classification accuracies using selected subsets of features, where the definitions of TN, TP, TP1, TP2 and ACC are given in Figure 8. Error bars in the chart indicate the corresponding standard errors for 30 modeling replicates.

The NCA feature selection algorithm was applied to the feature set of FI675-FI750 for two-class and three-class classification, respectively. Figure 9a shows the normalized feature weights derived from the feature selection algorithm for two-class classification. Both FI675 and FI750 provided many features that had large weights and could be considered strongly relevant to the CI detection of pickling cucumbers, which was also true in the case of three-class classification (not presented). With the features ranked by weight as shown in Figure 9b, it is conceivable to set the optimal subset of features by exhaustive enumeration of all the subsets of the ranked features, which, however, would be computationally prohibitive. Instead, the top 100 features were evaluated for building classification models by incrementally including one more feature from the highest to the lowest weight. From Figure 9b, the classification accuracy (on the test data) initially increased with the number of features and tended to plateau at a level on par with that obtained using a full set of features, despite the accuracy fluctuations with the further addition of features. Figure 9c shows the average classification accuracies for 30 modeling replicates. In the two-class classification, feature selection slightly improved TN, TP and ACC, and particularly ACC increased to 97.4%, which was significantly better than what was achieved using all the features (Figure 8). In the three-class classification, TN, TP2 and ACC also increased to a varying degree, while TP1 decreased and the improvement in ACC was statistically insignificant. The NCA feature selection algorithm allowed reducing the number of features from 334 to 54 and 75 on average for the two-class and three-class classification, respectively. Future work is, therefore, warranted to evaluate other feature selection algorithms that may achieve more aggressive feature reductions, while enhancing classification accuracies.

Classification results obtained using the full sets or downsized sets of features demonstrated that the dual-band CFI technique is a potentially viable tool for the detection of CI in pickling cucumbers. The feature fusion, which concatenates the features at different wavebands together, was found effective for boosting the model accuracy, especially in the more challenging three-class classification. Based on feature selection, the overall accuracy of 97.4% in the two-class classification compare favorably with those attained by using hyperspectral imaging for CI detection of cucumber fruit [12,13,14], and the accuracy of 91.3% in the three-class classification is comparable to 91.6% reported in [15]. Compared to hyperspectral imaging, the CFI technique acquires images at only two wavelengths, thus, significantly improving the imaging speed and reducing the data processing burden; moreover, it is more cost effective in system instrumentation. It should be noted that the camera exposure of the CFI system was set at 1 s for capturing fluorescent emissions, which is still too long for real-time inspection. Potential solutions for faster imaging are to use a more powerful excitation light source and a sensitive fluorescence detector with high quantum efficiency values at wavebands of interest. Using a standalone UV-blue lamp instead of the light source channel into a digital light projector for sample excitation could be a more cost-effective and light-efficient configuration. Lower accuracy in the three-class classification could be partly due to potential scoring errors in delimiting the two CI classes of pickling cucumbers. Furthermore, other modeling algorithms such as convolutional neural networks [35] should be considered.

## 4. Conclusions

This study demonstrated that dual-band chlorophyll fluorescence imaging (CFI) at 675 nm and 750 nm is an effective modality for the detection of chilling injury (CI) in pickling cucumbers. After chilling and post-chilling treatments, pickling cucumbers had developed symptoms such as skin pitting, water-soaked spots, browning and decay, which were manifested as darker areas in the fluorescence images. Image vignetting corrections improved the overall quality of fluorescence images. Linear support vector machine (SVM) models, built using the fusion of features at 675 nm and 750 nm extracted from the fluorescence images, resulted in overall accuracies of 96.9% and 91.2% in the two-class and three-class classification, respectively. The feature fusion was effective for significantly boosting model accuracies, especially for the three-class classification, compared to using the features for single wavelengths. Better results for the two-class and three-class classification were attained with overall accuracies of 97.4% and 91.3%, respectively, by selecting a subset of features for SVM modeling. Further research is needed, so that dual-band CFI can be used for real-time inspection of pickling cucumbers for chilling injury.

## Data Availability

Not applicable.
